# Increased MMP activity in curved geometries disrupts the endothelial cell glycocalyx creating a proinflammatory environment

**DOI:** 10.1371/journal.pone.0202526

**Published:** 2018-08-23

**Authors:** Scott Cooper, Alexander Emmott, Karli K. McDonald, Marc-Antoine Campeau, Richard L. Leask

**Affiliations:** 1 Department of Chemical Engineering, McGill University, Montréal, Quebec, Canada; 2 Montreal Heart Institute, Montréal, Quebec, Canada; Michigan Technological University, UNITED STATES

## Abstract

Wall shear stress gradients (WSSGs) induce an inflammatory phenotype in endothelial cells (ECs) which is hypothesized to be mediated by mechanotransduction through the EC glycocalyx (GCX). We used a three-dimensional *in vitro* cell culture model with a 180^o^ curved geometry to investigate if WSSGs created by curvature can cause EC inflammation and disruption of the GCX. The hydrodynamics of the model elicited a morphological response in ECs as well as a pattern of leukocyte adhesion towards the inner wall of curvature that was attenuated with enzymatic removal of GCX components. GCX degradation was also observed in regions of curvature which corresponded to increased activity of MMPs. Together, these results support the hypothesis that the EC GCX is involved in mechanotransduction of WSSGs and that components of the GCX are regulated by MMP activity in regions of curvature.

## 1. Introduction

The localization of atherosclerosis to regions of disturbed flow is hypothesized to be caused by endothelial cell (EC) dysfunction in response to the wall shear stress (WSS) patterns in these areas [1]. ECs are sensitive to WSS and are known to exhibit an anti-inflammatory phenotype in response to steady, uniform WSS and a pro-inflammatory phenotype, including cellular rounding and increased leukocyte adhesion, when exposed to wall shear stress gradients (WSSGs) [2–6]. Curved arteries exhibit WSSGs which are prone to focal inflammation and atherosclerosis [7].

The mechanotransduction of WSS is poorly understood in ECs, however, the apical glycocalyx (GCX) layer is commonly implicated [8, 9]. The GCX extends 0.5–4.5μm into the vessel *in vivo* [10–12] and is comprised of a variety of components including glycocsaminoglycans of which heparan sulfate (HS) is the most abundant [13]. It is a dynamic structure [14] whose disruption has been linked to atherosclerosis and diabetes [15–17]. Shedding of GCX components has been linked to matrix metalloproteinases (MMPs) expression and activity, which has been documented to increase in regions of complex flow both *in vivo* [18] and *in vitro* [19].

We investigated how the hydrodynamics of a curved vessel related to the inflammatory response of ECs and the regulation of GCX health. Using a novel *in vitro* cell culture model, it was observed that cell morphology and leukocyte adhesion patterns were dependent on GCX integrity and that enzymatic degradation of GCX components resulted in a loss of these observed patterns. We hypothesized that the hydrodynamic forces created by a curved geometry were responsible for regional GCX degradation which was linked to MMP regulation. Together these findings can explain the observed focal inflammation observed in regions of vessel curvature.

## 2. Materials and methods

### Three-dimensional cell culture models

**Fig 1:**
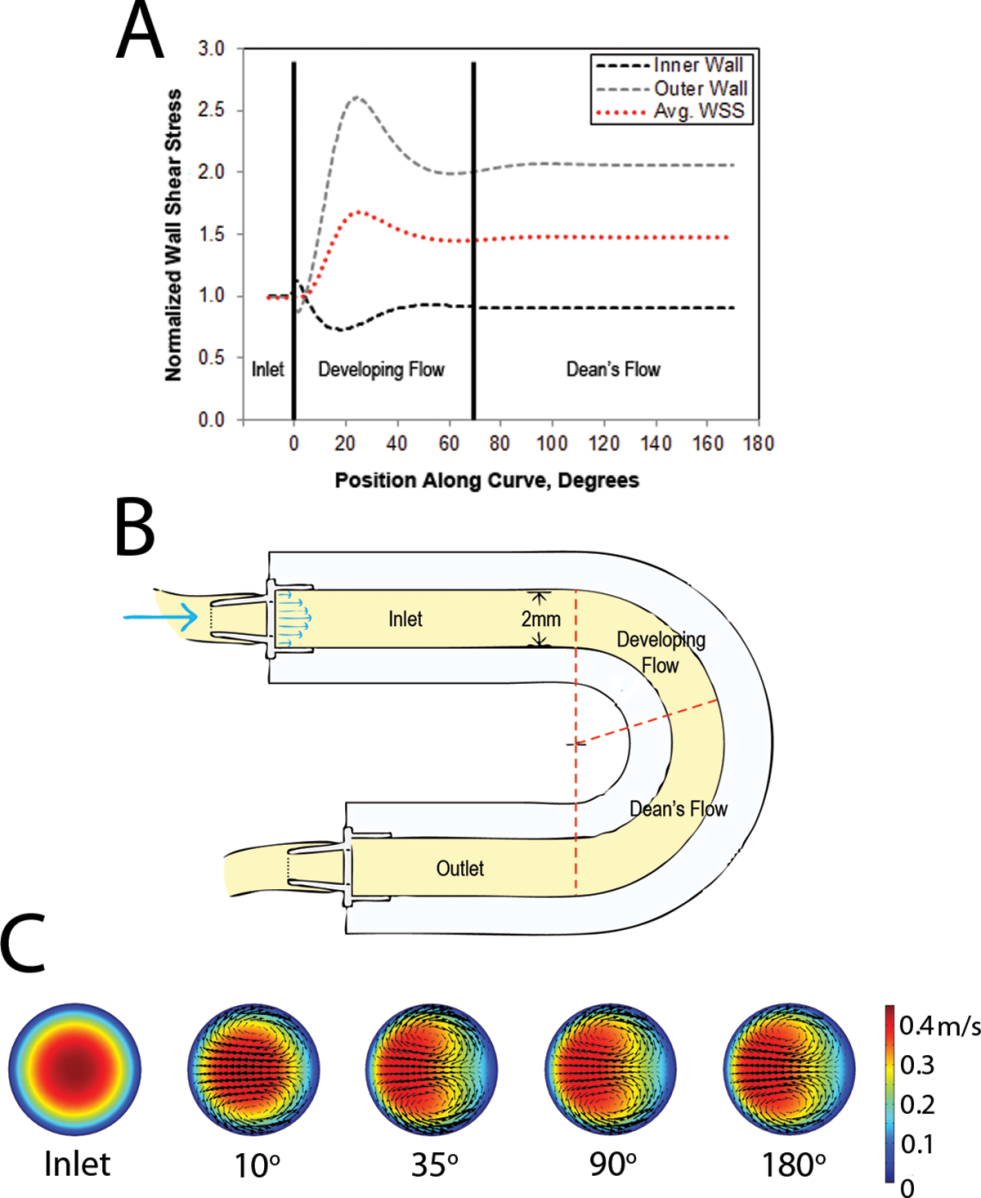
Overview of curved model and associated fluid dynamics. (A) WSS plot normalized to inlet WSS of 10 dyne/cm^2^ with 3 defined regions of curvature, (B) schematic drawing of 180^o^ curve model with regions of curvature annotated and (C) Cross-sectional velocity plots at different positions along the curve demonstrating secondary flows and fluid momentum shift (right: inner wall, left: outer wall).

*In vitro* models were made of Sylgard®184 (Dow Corning), prepared similarly to previously described methods [20]. A polished metal rod was bent to match the radius of curvature then cast in a mold with Sylgard®184. Once the elastomer was fully cured, the metal rod was removed and connectors for perfusion tubing were added to the inlet and outlet of the model. Dimensionless analysis matched the model dynamics to regions of arterial curvature. The Dean’s Number (D_N_) is the most relevant of these parameters and is defined as:

DN=ReN×[D/2RCurv]12

which takes into account the Reynolds Number (Re_N_) and the curvature ratio (D/2R_Curv_) of a curved cylindrical vessel [21]. The model had a vessel diameter (D) of 2 mm and a radius of curvature (R_Curv_) of 25.4 mm and the cell culture media had a viscosity and density of 9.75x10^-4^ Pa∙s and 994.3 kg/m^3^, respectively. By controlling the media flow rate, a Dean’s Number of 104 was achieved which falls in the physiological range (10–700 *in vivo*, [22]) and corresponds with a relevant inlet WSS of 10 dyne/cm^2^ and Reynolds Number of 543. Under these conditions, fluid momentum caused differential WSS profiles between the inner and outer wall of curvature, Fig 1A.

The WSS profile was used to segment the model into 3 distinct flow regions for analysis: the uniform WSS inlet region (inlet), a developing flow region (developing flow) and a fully developed Dean’s flow region (Dean’s flow), Fig 1B.

### Computational fluid dynamics

CFD simulations were conducted in Comsol Multiphysics 5.0 (Comsol Inc. Burlington, MA) to determine the WSS profile in the cell culture model. Simulations were run at an inlet Reynolds number of 543 to achieve an inlet WSS of 10 dyne/cm^2^ and a Dean’s Number of 104. Wall effects from cells were assumed to be negligible due to their much smaller size compared to the channel diameter. WSS was calculated from the near wall velocity gradient using a mesh independent laminar solver for steady, incompressible, Newtonian flow with a zero-pressure outlet condition. Simulations also confirmed the development of secondary flows in the form of Dean vortices exhibiting transverse shear gradients along the model circumference, Fig 1C.

### Tissue culture

Human abdominal aortic endothelial cells (HAAECs, Coriell, AG09799) were cultured and grown to confluence in 0.1% gelatin-coated T-175 flasks in incubators (37°C, 100% humidity and 5% CO_2_) over 48 hours. The HAAECs were grown in EC media (PromoCell, C-22010) with 10% fetal bovine serum (Invitrogen, 26140–079) and 1% penicillin-streptomycin (Invitrogen, 15140–122).

The Sylgard®184 models were coated with 40 μg/mL fibronectin (Sigma Aldrich, F2006-5X5MG) for 24 hours prior to cell seeding. The cultured HAAECs were removed from the T-175 flasks using a trypsin solution (0.25% Trypsin/EDTA, Invitrogen) and seeded into the *in vitro* models at a density of 1x10^6^ cells/mL. The HAAECs were cultured for 48 hours prior to each flow experiment, with a fresh media change after 24 hours, allowing them to establish a confluent monolayer on the models’ luminal surface.

NB4 cells (DSMZ, ACC 207), a human promyelocytic cell line, were used to simulate leukocyte adhesion to the endothelium [23, 24]. NB4 cells were cultured in suspension in T-75 flasks with RPMI Media (Global Cell Solutions, 89140–464) containing 10% fetal bovine serum (Invitrogen, 26140–079) and 1% penicillin-streptomycin (Invitrogen, 15140–122). Prior to experimentation, NB4 cells were treated with RPMI media spiked with 10^−6^ M all-trans retinoic acid (ATRA) (Life Sciences, 89158–732) for 48 hours to differentiate them into neutrophil-like cells exhibiting increased expression of β1 and β2 integrins, as previously described [25]. Following ATRA stimulation, the NB4 cells were suspended in HAAEC media at a cell density of 1.67x10^6^ cells/mL and then used in adhesion assays.

### HS degradation

To impair the structure of the GCX, degradation of HS was performed by heparinase III (Sigma Aldrich, H8891-10UN) treatment [8, 26]. Briefly, serum free media (PromoCell, C-22010) was spiked with 180mU/mL heparinase III and incubated with ECs for 2 hours immediately prior to perfusion experiments. This resulted in a significant decrease in HS intensity of 33±4%, with no loss in cell viability, as previously shown [27]. At the start of experiments, standard EC media was reintroduced into the models.

### Perfusion experiments

**Fig 2:**
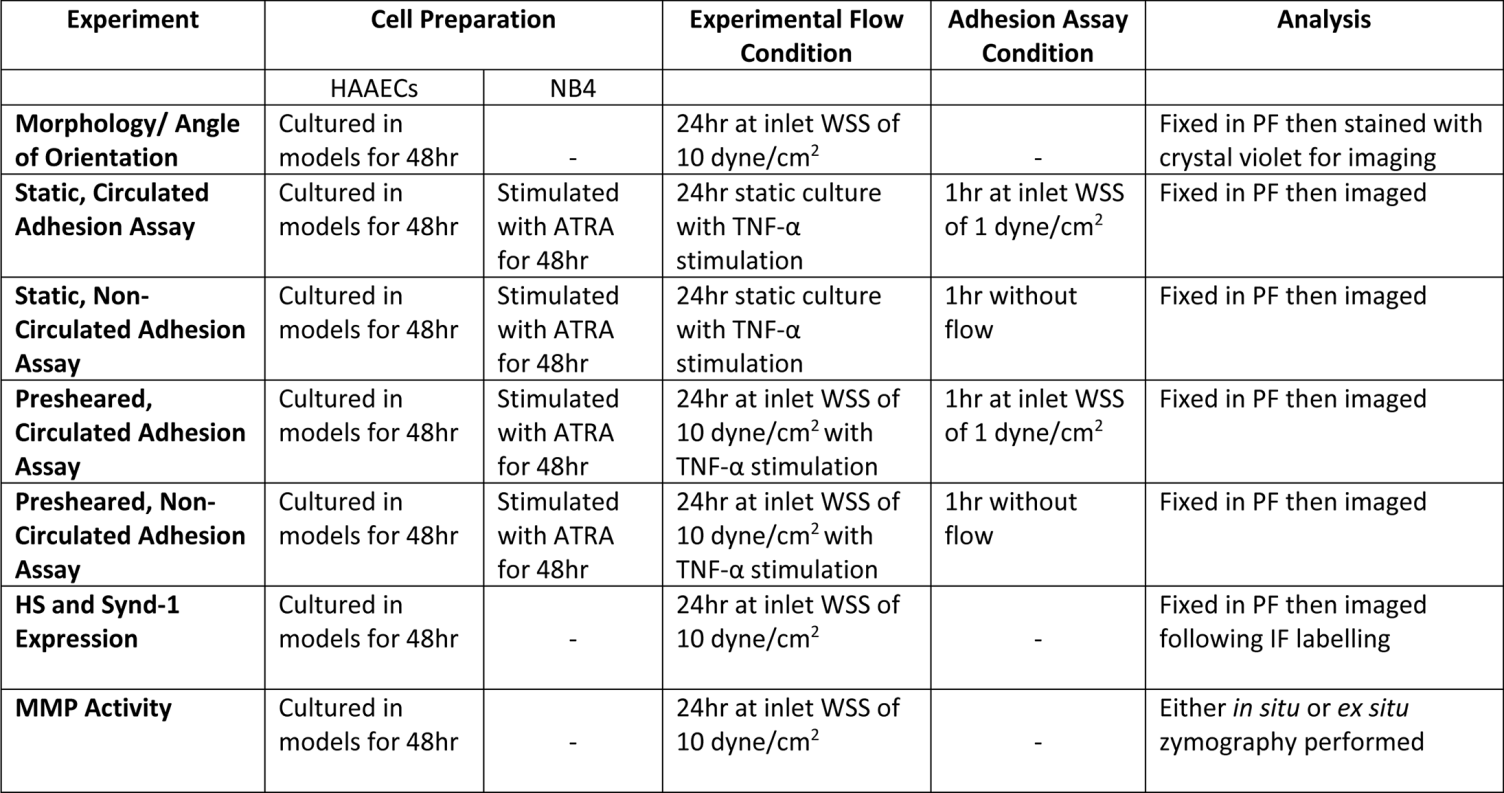
Summary of experimental conditions and subsequent assay analysis. When HS degradation was included in an experiment, this would be preformed immediately prior to the appropriate “Experimental Flow Condition”.

Perfusion experiments were performed in an incubator at standard cell culture conditions (37°C, 100% humidity and 5% CO_2_) in a sterilized closed-loop as previously described [20]. The flow rate was maintained to achieve an inlet WSS of 10 dyne/cm^2^ in morphology and preshearing experiments and 1 dyne/cm^2^ in circulating adhesion assays. A summary of the different experimental protocols can be referred to in Fig 2.

### EC morphology

The shape index (SI), as defined by Nerem *et al* ($SI=\frac{4\pi *Area}{{Perimeter}^{2}}$) [4], was determined by staining ECs with Crystal Violet and imaging the 3 defined sections of the *in vitro* model. The images were processed by a MatLab® protocol to determine the SI of the stained nuclei of at least 10 cells per image [20]. Cells become more rounded as the SI approaches 1 and more elongated as it approaches 0.

### Immunofluoresence quantification of glycocalyx

Immediately following the completion of an experiment, ECs were fixed with a 1% paraformaldehyde solution and subsequently probed with a monoclonal antibody specific to HS (Millipore, MAB2040) or syndecan-1 (Santa Cruz, SC-7099) and an Alexa Fluor® 488 secondary antibody (Invitrogen, A31570). Images were taken with a laser scanning confocal microscope (Zeiss Exciter) and an average intensity of at least 10 cells per image was used.

### NB4 adhesion assays

Leukocyte adhesion to the endothelium was investigated using ATRA-stimulated NB4 cells. NB4 cells exhibit similar binding sites to leukocytes following ATRA stimulation and have thus been used in similar adhesion studies [23, 25, 28]. Models were presheared for 24 hours at an inlet WSS of 10 dyne/cm^2^ and were simultaneously stimulated with 10 μg/mL of the cytokine TNF-α to increase total adhesion [29]. Following this treatment, a suspension of 1x10^6^ NB4 cells/mL was either statically added to the models (non-circulated) or circulated at an inlet WSS of 1 dyne/cm^2^ [26, 30]. Both the low WSS circulation and TNF-α stimulation were included in adhesion assays to ensure measurable differences in cell adherence over the time scale accessible *in vitro*. Following a 1 hour exposure to the NB4 cell suspension, the models were fixed using a 1% paraformaldehyde solution and imaged. Images included the entire diameter of the model and the average of 3 images were taken for each data point.

### *In situ* gel zymography

HAAECs were fixed in 1% paraformaldehyde and rinsed with PBS. DQ gelatin (Molecular Probes, D12054) was diluted in Tris Zymo buffer (50mM Tris (pH 7.3), 15mM CaCl_2_) at a concentration of 1:40. Cells were incubated with the solution for 16hr then washed and mounted before imaging.

### *Ex situ* gel zymography

Following experiments, HAAECs were removed from the models with a 0.25% Trypsin solution (0.25% Trypsin/EDTA, Invitrogen) and were treated with RIPA lysis buffer. Samples were prepared and run on acrylamide gels as previously described [31].

### Statistical analysis

All results were expressed as mean ± standard error of the mean and experiments were performed at least in triplicates. Analysis of results was completed using Graphpad Prism 5 (Graphpad Software, La Jolla, CA) software. One-way and Two-way ANOVAs were used for comparisons when needed and were accompanied by multiple comparisons tests (Bonferroni post-hoc tests). Differences between means were considered significant at P<0.05.

## 3. Results

### Characterization of fluid dynamics of the *in vitro* model using CFD analysis

The WSS profile was obtained from CFD analysis using the experimental flow conditions, Fig 1. This profile was used to section the model into 3 regions where distinct WSS patterns were observed. The inlet demonstrated a uniform parabolic velocity profile and was used as an internal control. The developing flow region was defined from 0 to approximately 70^o^ around the curve where the velocity profile changed in the axial direction. WSS gradients were observed on the inner and outer wall of curvature in this region. The Dean’s flow region from 70^o^ to the curve outlet exhibited fully developed Dean’s flow with a lower WSS on the inner wall of curvature which was similar in magnitude to the inlet and no spatial gradients in the axial direction.

### Morphological response of HAAECs to WSS

**Fig 3:**
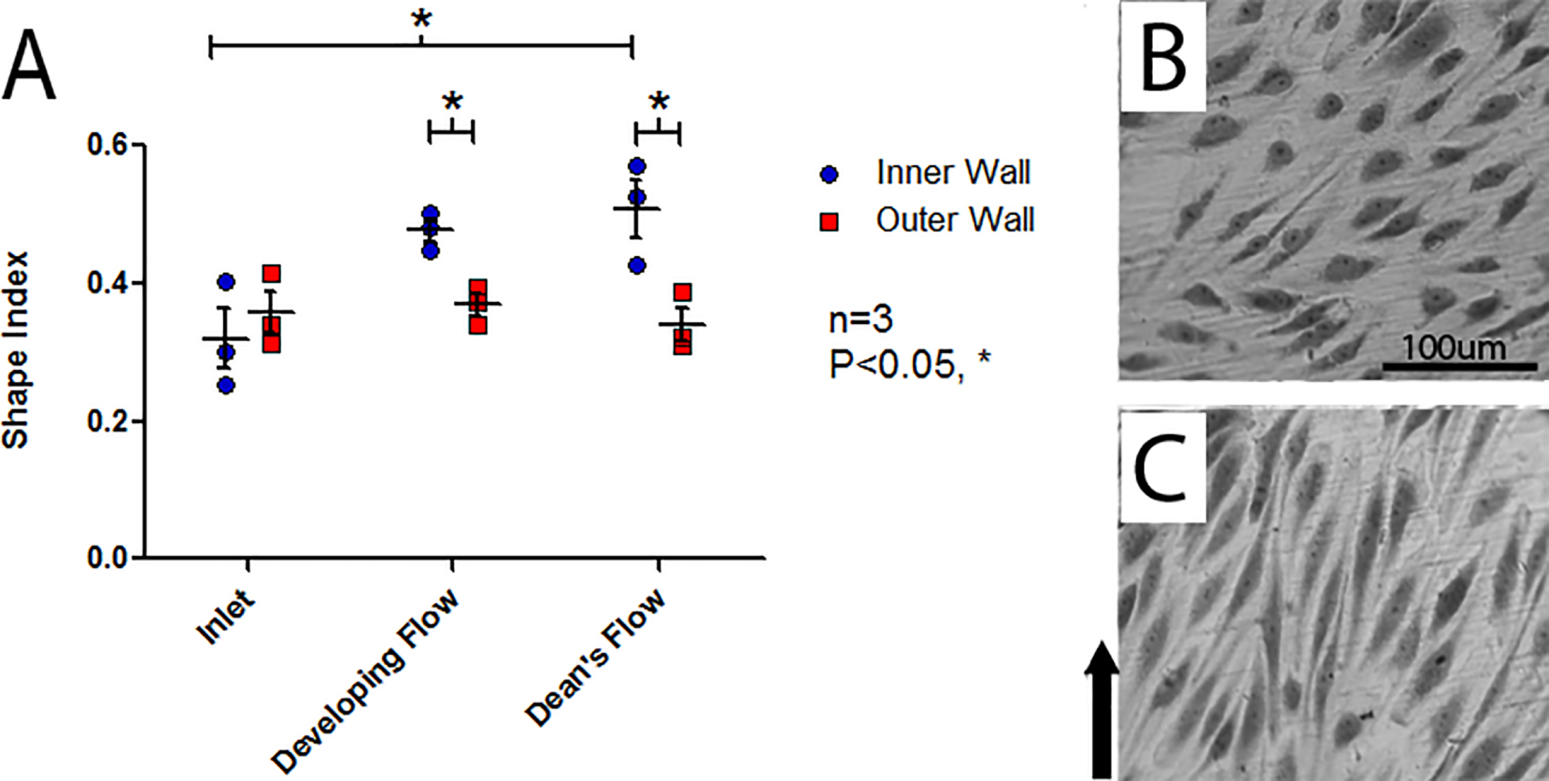
Analysis of shape index of HAAECs. (A) Analysis of shape index of HAAECs on the inner and outer walls of curvature following 24hr of flow conditioning at an inlet WSS of 10dyne/cm^2^ (n = 3, mean SI ± SEM). The inner wall in the Dean’s flow region had a significantly higher SI than the inlet inner wall (Bonferroni post-hoc test, P<0.05). The developing and Dean’s flow regions also exhibited a significantly higher SI on the inner wall compared to the outer wall in the same region (Bonferroni post-hoc test, P<0.05 and P<0.05, respectively). Representative images used for analysis from Developing Flow region, with direction of flow indicated, in the (B) inner wall of curvature (average shape index = 0.51) and (C) outer wall of curvature (average shape index = 0.34) with direction of flow identified with an arrow.

Following flow conditioning for 24 hours at an inlet WSS of 10 dyne/cm^2^, a significant dependence of EC morphology (SI) to both the side of the curve (two-way ANOVA, P<0.01) and model region (two-way ANOVA, P<0.05) were observed, Fig 3 (S1 Table). There was also significant interaction between model region and side of the curve (two-way ANOVA, P<0.05). In the Dean’s flow region, the inner wall of curvature demonstrated significant rounding when compared to the inlet (Bonferroni post-hoc test, P<0.05). Further, the inner wall of the developing and Dean’s flow regions exhibited significant rounding when compared to their matched outer wall (Bonferroni post-hoc test, P<0.05 and P<0.05, respectively).

### Angle of orientation of HAAECs in response to WSS

**Fig 4:**
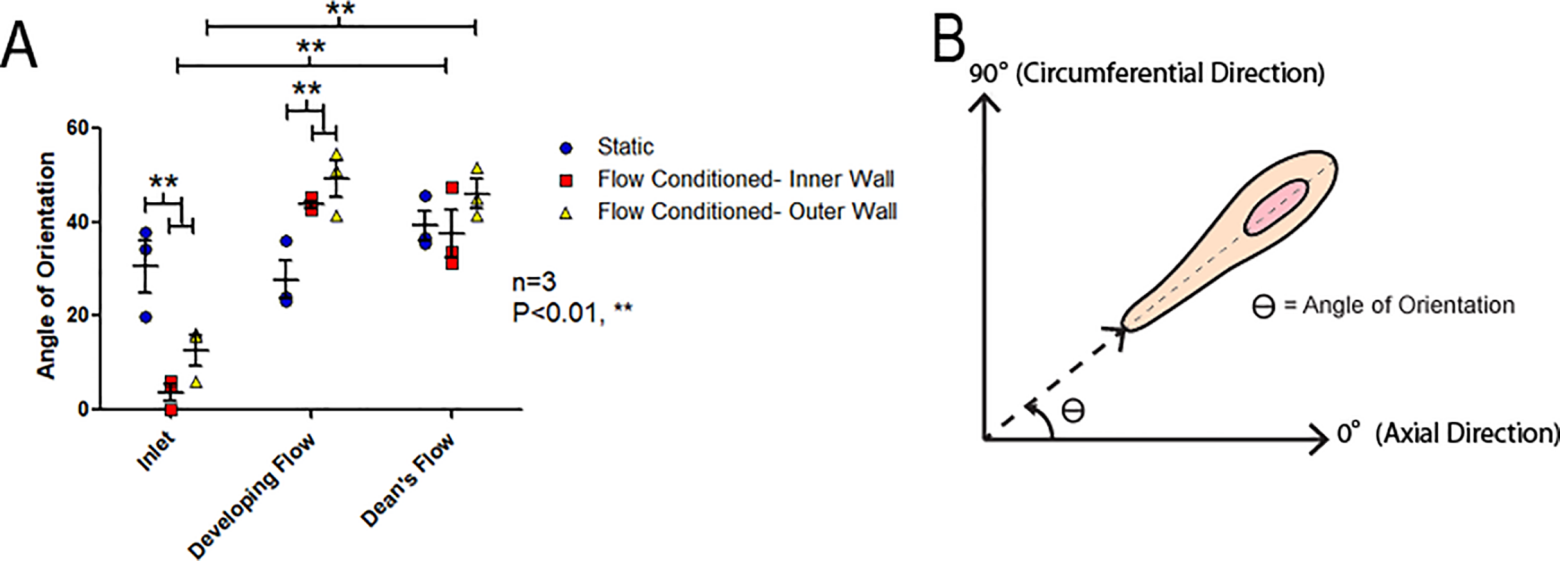
Angle of orientation of HAAECs. (A) Angle of orientation (absolute value) of HAAECs on the inner and outer walls of curvature following 24hr of flow conditioning at an inlet WSS of 10dyne/cm^2^ (n = 3, mean angle ± SEM). Both the inner and outer walls of the inlet were significantly more oriented in the direction of axial flow when compared to static controls (Bonferroni post-hoc test, P<0.01), however, flow conditioned HAAECs were significantly less oriented across the longitudinal axis in the developing flow region (Bonferroni post-hoc test, P<0.01). In both the inner and outer wall of the Developing and Dean’s flow regions, HAAECs were significantly less oriented along the vessel axis when compared to their respective inlet control (Bonferroni post-hoc test, P<0.01). (B) Representative cartoon of the angle of orientation (θ) of the longitudinal cell axis with 0^o^ being axial and 90^o^ being circumferential to the channel.

Following flow conditioning for 24 hours at an inlet WSS of 10 dyne/cm^2^ (flow conditioned), the angle of orientation, defined as the absolute value of the angle of the longitudinal cell axis relative to the axial flow through the channel (0^o^ is perfectly along the axial direction and 90^o^ is perfectly circumferential), was significantly affected by the model region as well as the side of the curve (two-way ANOVA, P<0.001 and P<0.05, respectively), Fig 4 (S2 Table). Both the inner and outer walls of the inlet region in presheared models demonstrated HAAECs which were significantly more oriented along the longitudinal axis of the model than static controls (Bonferroni post-hoc test, P<0.01). However, ECs in the developing and Dean’s low regions were less oriented along the longitudinal axis than static controls (Bonferroni post-hoc test, both P<0.01). In both the inner and outer walls of presheared models, the respective inlet was significantly more oriented along the longitudinal axis of the model than the developing and Dean’s flow regions (Bonferroni post-hoc test, P<0.01).

### Distribution of adhered NB4 cells

Circulated and non-circulated adhesion assays were performed on both statically cultured and flow conditioned (presheared) HAAECs, with and without GCX degradation. Adhesion assays on statically cultured HAAECs were included to determine if any bias was attributed to the hydrodynamics in the model. Since the HAAECs were not exposed to the shear fields created in the model, there would be no phenotypical changes in the cells and hence any patterns of adhesion could be attributed to hydrodynamics acting on the NB4 cells.

**Fig 5:**
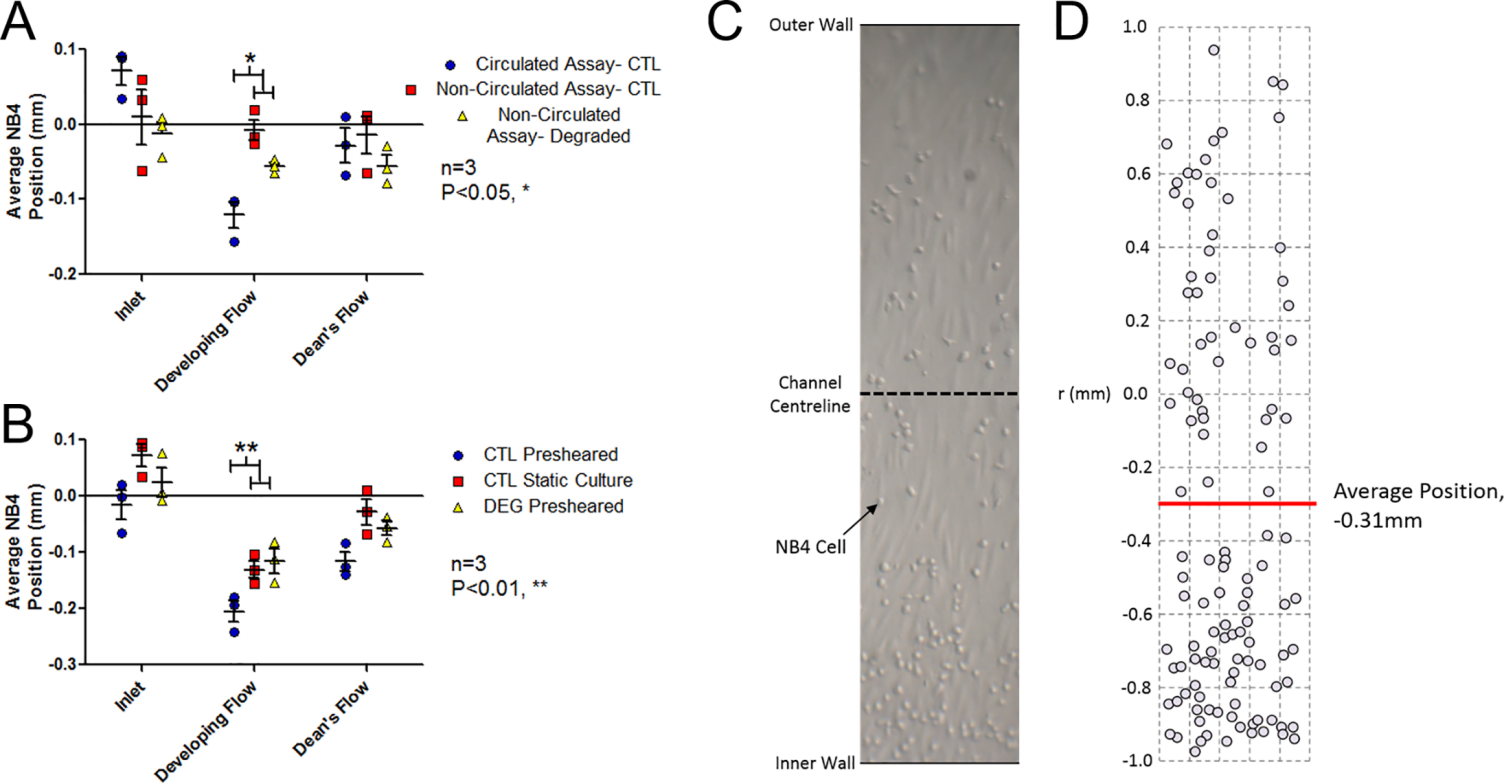
Average position of adhered NB4s relative to the centreline with the inner wall in the negative direction and outer wall in the positive direction. (A) Both circulated (inlet WSS of 1 dyne/cm^2^) and non-circulated (no WSS) adhesion assays for statically cultured control (CTL) and HS-degraded (DEG) HAAECs (n = 3, mean average position ± SEM). The developing flow region of the circulated adhesion assay showed a significant bias towards the inner wall compared to static controls (Bonferroni post-hoc test, P<0.05). (B) Mean average position of circulated (inlet WSS of 1 dyne/cm^2^) adhesion assays following 24hr preshearing (inlet WSS of 10 dyne/cm^2^) or static culture. Controls exhibited a significant bias in adhesion to the inner wall in the developing flow region relative to HS-degraded HAAECs (Bonferroni post-hoc test, P<0.01). (C) Representative image of the full model diameter used for analysis from the Dean’s Flow region of circulated adhesion assay of CTL presheared HAAECs. (D) Analysis of the image collecting coordinates of each adhered NB4 cell to determine average position as reported in (A) and (B), demonstrating a bias to the inner wall of curvature.

Static cultures demonstrated that the type of assay (circulated/ non-circulated) and model region both had a significant effect on adhesion (two-way ANOVA, P<0.05 and P<0.05), Fig 5A (Table A in S3 Table). In the developing flow region, there was a significant bias of adhesion towards the inner wall of curvature in circulated compared to non-circulated assays. There was no significant difference in adhesion distribution between control and degraded cultures.

The adhesion assays were then repeated following flow conditioning for 24 hours at an inlet WSS of 10 dyne/cm^2^ (presheared). Both degradation and location had a significant effect on adhesion (two-way ANOVA, P<0.001 and P<0.001, respectively), Fig 5B (Table B in S3 Table). Following preshearing, adhesion was higher on the inner wall of curvature in the developing flow region when compared to statically cultured control (Bonferroni post-hoc test, P<0.01) and HS-degraded (Bonferroni post-hoc test, P<0.01) HAAECs.

### HS and syndecan-1 expression

**Fig 6:**
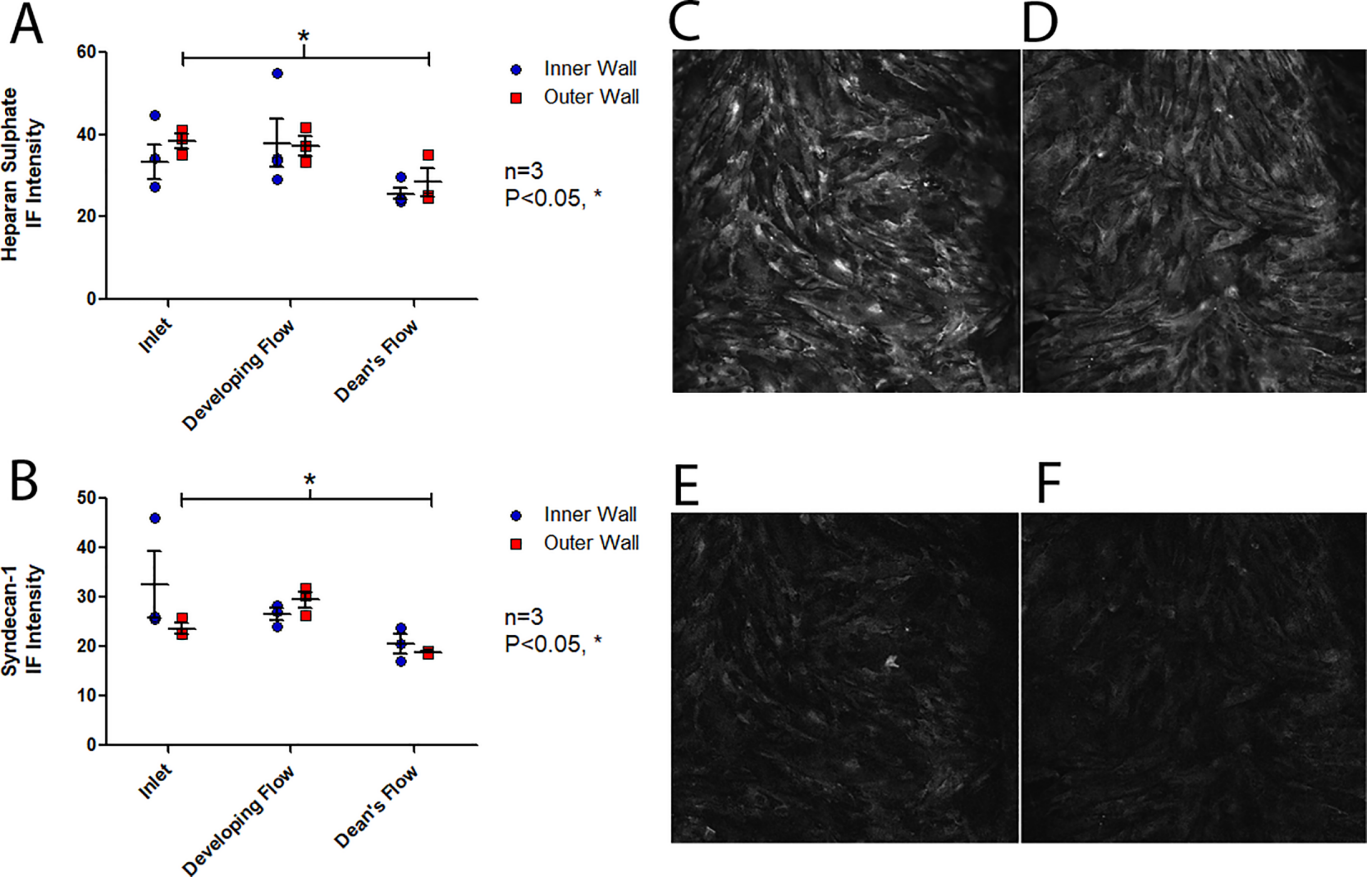
Immunofluorescence staining of HS and Syndecan-1. Following 24hr of flow conditioning (inlet WSS of 10 dyne/cm^2^), mean fluorescence intensity (n = 3, mean intensity ± SEM) of (A) of HS which showed a significant decrease in mean intensity in the Dean’s flow region relative to the inlet (Bonferroni post-hoc test, P<0.05) and (B) Syndecan-1 which also showed a significant decrease in mean intensity in the Dean’s flow region relative to the inlet (Bonferroni post-hoc test, P<0.05). Representative images of (C) Inlet and (D) Dean’s Flow HS immunofluorescence for the outer wall of curvature and (E) Inlet and (F) Dean’s Flow Synd-1 immunofluorescence for the outer wall of curvature.

Following flow conditioning for 24 hours at an inlet WSS of 10 dyne/cm^2^, the expression of HS was similar between the inner and outer wall, however there was significant variation in HS amongst the model regions (two-way ANOVA, P<0.01), Fig 6A (Table A in S4 Table). A significant decrease in HS expression was observed in the Dean’s flow region when compared to the inlet (Bonferroni post-hoc test, P<0.05).

Similarly, following flow conditioning for 24 hours at an inlet WSS of 10 dyne/cm^2^, the side of the curve showed no effect on syndecan-1 expression, however expression varied significantly by model region (two-way ANOVA, P<0.01), Fig 6B (Table B in S4 Table). On the outer wall of curvature, there was a significant decrease in syndecan-1 expression in the Dean’s flow region when compared to the inlet (Bonferroni post-hoc test, P<0.05).

### MMP activity

**Fig 7:**
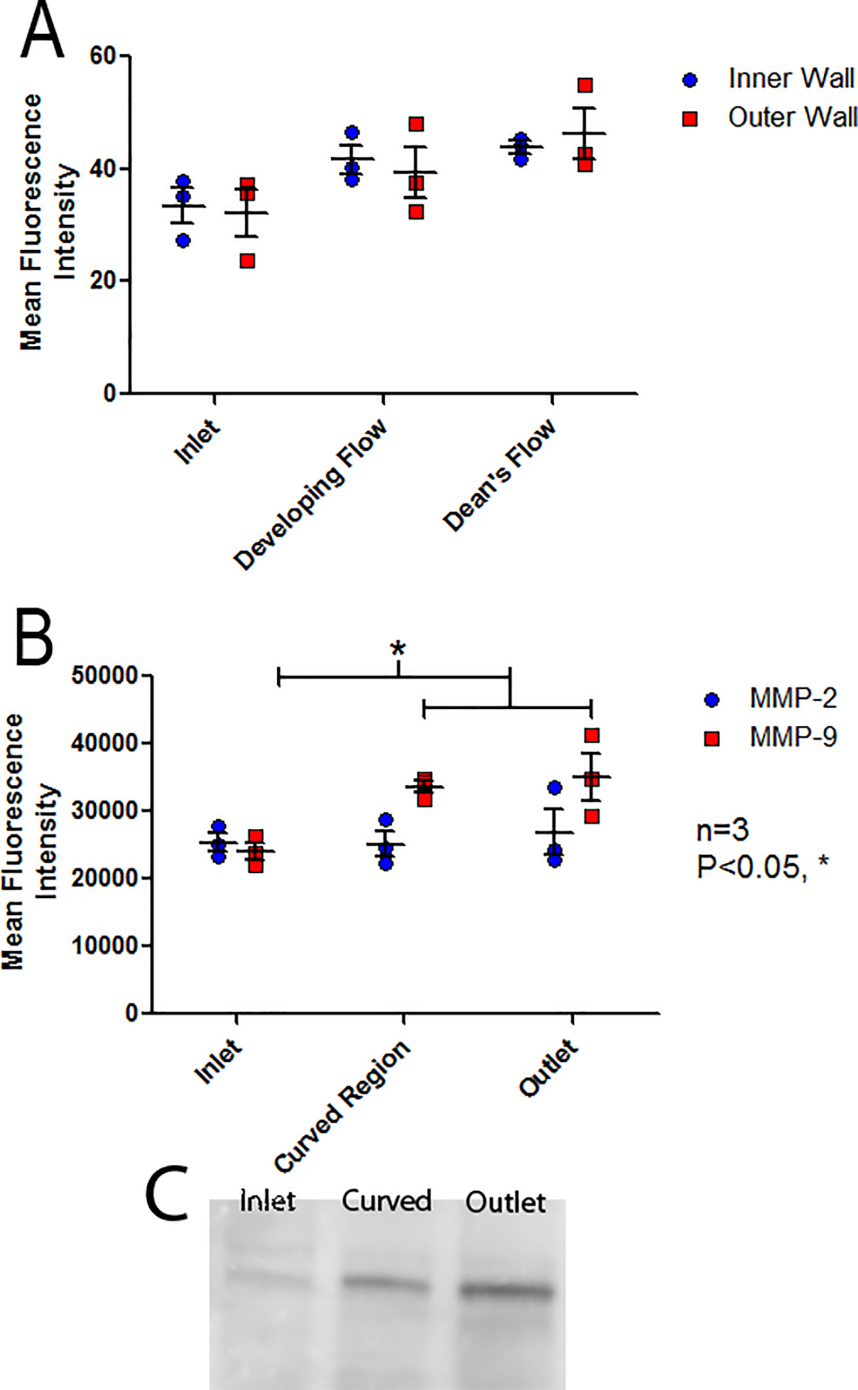
Analysis of MMP activity throughout the curved model. (A) Regional MMP activity quantified by *in situ* gel zymography following treatment of HAAEC with DQ gelatin showing a trend for increased activity throughout the curve and (B) Regional MMP activity quantified by *ex situ* gel zymography showing a significant increase in MMP-9 activity in the curve and outlet regions of the model when compared to the inlet (Bonferonni post-hoc test, P<0.05). (n = 3, mean intensity ± SEM). (C) Representative zymography image from *ex situ* MMP-9 experiments.

Following flow conditioning for 24 hours at an inlet WSS of 10 dyne/cm^2^, *in situ* gel zymography demonstrated that MMP activity was significantly affected by model region (two-way ANOVA, P<0.05) but did not show dependence on the side of curvature, Fig 7A (Table A in S5 Table).

*Ex situ* gel zymography was performed and HAAECs were pooled from both the Developing Flow and Dean’s Flow regions and labelled “Curved region” to ensure adequate protein concentrations for analysis. *Ex situ* gel zymography demonstrated that MMP activity was dependent on model region (one-way ANOVA, P<0.05). Significantly higher MMP-9 activity was observed in both the curve and the outlet when compared to the inlet of the model (Bonferroni post-hoc test, P<0.05), whereas no differences were observed for MMP-2, Fig 7B (Table B in S5 Table).

## 4. Discussion

In this study, we investigated the role of the EC GCX in cellular inflammation in regions of a 180^o^ curved vessel. Results demonstrated that the hydrodynamics of this geometry regionally degraded the EC GCX which correlated with increased MMP activity and altered patterns of leukocyte adhesion.

CFD simulations suggested a spatially variable midline shear field exhibiting WSSGs along the model. Further, 3D velocity profiles demonstrated recirculating Deans vortices, creating secondary gradients across the circumference of the model. The fluid dynamics of the model were found to significantly alter EC phenotype, with observed differences in morphology (quantified by shape index and orientation) and leukocyte adhesion patterns in the different model regions. Degradation of HS and syndecan-1 was observed downstream of WSSGs, corresponding to regions of increased MMP activity, suggesting a link between GCX health and MMP regulation in curved vessels.

### HAAECs exhibited a differential morphological response to WSS in regions of curvature

The model’s fluid dynamics lead to an elongated morphology on the outer wall of curvature, with the developing and Dean’s flow regions exhibiting significantly greater elongation than both the inlet and matched inner wall of the model, Fig 3. This can be correlated with a higher WSS on the outer wall due to momentum carrying the bulk flow to the outer curve. Further, this resulted in a lower WSS acting on the inner wall of curvature that was accompanied by secondary flows. Elongation of EC morphology is indicative of an "atheroprotective" phenotype [5], indicating the outer wall may be comparatively less prone to developing pathology than the more rounded ECs on the inner wall as seen in animal models [32, 33].

Potter *et al* used an elongation index to show that ECs on the outer wall of curvature were elongated when compared to those found on the inner wall of porcine aortic ECs in tissue culture models [34]. In contrast, Wang *et al* found increases in VCAM-1 and E-selectin, markers of EC inflammation, on the outer wall of curvature, but these results were confounded by having suspended red blood cells which they hypothesized “bombarded” the outer wall, causing more severe EC damage [35]. These results support the former findings, suggesting the fluid dynamics in a curved vessel result in a differential EC response favouring an inflamed, rounded shape, on the inner wall of curvature.

The angle of EC orientation demonstrated significantly less alignment with the direction of bulk flow on both sides of the flow channel in the developing flow region when compared with static controls, Fig 4. Typically, WSS will result in alignment with the direction of bulk flow in regions of steady, uniform flow [5]. In contrast, complex fluid dynamics and WSSGs have been shown to cause a less organized orientation [5]. Interestingly, there was low variance in the angle of orientation in the curve, suggesting the cells were in relative alignment but this alignment was skewed towards the circumferential direction. This lack of orientation in the axial direction can be attributed to the nature of Dean's flow. Dean's flow produces secondary flows in the circumferential direction which redistribute the increased momentum on the outer wall back towards the inner wall of curvature, as shown in Fig 1C. Therefore, this flow will take a more circumferential than axial path when compared to flow in a straight cylinder. The angle of orientation may have been offset from the axial direction due to these secondary flows. Potter *et al* showed that *in vivo*, ECs in the aortic arch had increased variability in alignment when compared to static *in vitro* cultures [36]. Ghriallais *et al* found an increased alignment in the direction of flow compared to static controls in a curved model (without inner/ outer wall specificity) and noted less alignment in curved regions when compared to straight tube models [37]. Alloush *et al* observed decreased alignment with flow in regions of curvature where complex fluid dynamics were present [38]. This study furthers these findings suggesting the orientation of the ECs may be a consequence of secondary flows.

### Differential NB4 adhesion to ECs was observed in regions of curvature

To determine the effect of the model’s hydrodynamics on adhesion patterns, ECs were statically cultured before being exposed to a suspension of either circulated or non-circulated NB4 cells for 1hr. Circulated adhesion assays were performed to better mimic the rolling and tethering mechanics which accompany leukocyte adhesion *in vivo* but at a low enough WSS that it would not illicit a phenotypical response in ECs. Therefore, any adhesion patterns which differed between the circulated and non-circulated adhesion assays were assumed to be the result of the hydrodynamics acting on the NB4 cells. These experiments demonstrated the hydrodynamic forces caused a bias in NB4 adhesion towards the inner wall of curvature in the developing flow region, Fig 5A.

To determine the phenotypical response of ECs to curved vessel hydrodynamics, ECs were presheared (flow conditioned for 24 hours at an inlet WSS of 10 dyne/cm^2^) prior to circulated adhesion assays. These assays demonstrated a significant bias of NB4 adhesion to the inner wall of curvature beyond the effect of the hydrodynamic forces acting on the circulated NB4 cells, Fig 5B. This bias can therefore be attributed to the WSS fields eliciting a phenotypical change in ECs in these regions. Suo *et al* demonstrated an increased expression of cellular adhesion molecules on the inner wall of curvature in mouse aortas which could help support this finding [39]. Similarly, increased adhesion has been linked to regions of WSSGs and lower WSS [40] and is indicative of EC inflammation. The lower observed WSS and secondary flows creating circumferential WSSGs on the inner wall of curvature, Fig 1, would agree with this focal inflammation. The observed adhesion patterns in this study are further supported by clinical evidence of an increased prevalence of plaque development on the inner wall of curvature in arteries [41–43].

### Disruption of the GCX attenuates NB4 adhesion patterns in regions of curvature

Mechanotransduction of fluid forces through the GCX was hypothesized to be responsible for the WSS mediated phenotypical changes which lead to the NB4 adhesion bias. Supporting this hypothesis, presheared HS-degraded ECs did not exhibit the amplified adhesion bias to the inner wall of curvature seen in control cultures, Fig 5B. Cooper *et al* demonstrated a similar effect in a 50% stenosis model, where non-uniform regional NB4 adhesion patterns were attenuated following GCX degradation [26].

### Disruption of HS and syndecan-1 occurs in regions of curvature

In the Dean’s flow region, significant decreases were observed in both HS and Syndecan-1 compared to the inlet, Fig 6, demonstrating that flow through regions of curvature negatively affected EC GCX health. Studies have found similar findings *in vivo*, reporting that GCX components, including HS, are focally shed in regions of disturbed flow [44–46]. The observed degradation was hypothesized to be the result of increased MMP activity in response to the complex of flow in the curved model. Studies utilizing oscillatory flow found upregulation of MMP-9 [47], bifurcation models increased activity of MMP-2 and MMP-9 in regions of WSSGs [19] and MMP expression has been linked to NF-κB activity which is regulated in response to WSSGs [36, 48, 49], all supporting this notion. A variety of MMPs have been linked to GCX disruption, with MMP-9 cleaving HS [50] and MMP-1 and MMP-14 cleaving syndecan-1 [51, 52].

### MMP activity was increased in regions of curvature

MMP activity was quantified by *in situ* and *ex situ* gel zymography, both demonstrating a correlation with MMP activity and GCX degradation. Although increased activity of the gelatinases (MMP-2 and MMP-9) was not observed in any distinct region with *in situ* analysis, there was still a significant overall effect of region on MMP activity. *Ex Situ* zymography showed significant increases in MMP-9 activity in the curve and outlet when compared to the inlet. Due to constraints in protein concentrations extracted from the models, ECs from the entire curve had to be pooled for this analysis, losing spatial resolution in this finding. However, observing this increase in the curve still correlated with decreased HS and syndecan-1 expression in the Deans Flow region which makes up the majority of the 180^o^ curve and was in agreeance with previous findings [49]. These results support the proposed hypothesis that the complex flow which develops in a curved geometry increases MMP activity which then degrades the GCX, eliciting an inflammatory response in ECs.

Although increased activity was expected in the curve where complex flow was found, it was unexpected to see higher MMP-9 activity in the outlet region where uniform flow was re-established. We hypothesize this to be a “wash down” effect whereby MMP-9 activation in the curve is subsequently carried downstream by the fluid flow. We have suggested a similar mechanism in a 50% stenosis model [26].

Overall, this study demonstrated that a curved vessel geometry elicits a differential inflammatory response in ECs. Specifically, experiments showed changes in morphology and NB4 adhesion patterns were tied to GCX health. Regional degradation of GCX components also correlated with increased MMP activity, suggesting MMP regulation in areas of complex flow can significantly affect GCX health and lead to an inflammatory EC response.

## Supporting information

S1 Table Shape index data for Fig 3. Raw data from morphology experiments to determine shape index in model regions.
(TXT)

S2 Table Angle of orientation data for Fig 4. Raw data from flow experiments to determine angle of orientation in model regions.
(TXT)

S3 Table Average position of NB4 adhesion for Fig 5. Raw data from adhesion experiments to determine position of NB4 adhesion in model regions.
(TXT)

S4 Table **Immunoflourescence intensity for Table A) Heparan sulphate and Table B) Syndecan-1 for Fig 6.** Raw data from flow experiments to determine abundance of heparan sulphate and syndecan-1 in model regions.
(TXT)

S5 Table **Table A) In situ gel zymography and Table B) Ex situ gel zymography for Fig 7.** Raw data to determine levels of MMP activity in the model regions.
(TXT)
